# *In silico* analysis of a therapeutic target in *Leishmania infantum*: the guanosine-diphospho-D-mannose pyrophosphorylase

**DOI:** 10.1051/parasite/2012191063

**Published:** 2012-02-15

**Authors:** S. Pomel, J. Rodrigo, F. Hendra, C. Cavé, P.M. Loiseau

**Affiliations:** 1 Université Paris-Sud 11, Faculté de Pharmacie, UMR 8076 CNRS, Chimiothérapie Antiparasitaire 5, rue Jean-Baptiste Clément 92296 Châtenay-Malabry France; 2 Université Paris-Sud 11, Faculté de Pharmacie, UMR 8076 CNRS, Conception et Synthèse de Molécules à Activité Thérapeutique Châtenay-Malabry Cedex France

**Keywords:** *Leishmania infantum*, GDP-mannose pyrophosphorylase, antileishmanial activity, *Leishmania infantum*, GDP-mannose pyrophosphorylase, antileishmanien

## Abstract

Leishmaniases are tropical and sub-tropical diseases for which classical drugs (*i.e.* antimonials) exhibit toxicity and drug resistance. Such a situation requires to find new chemical series with antileishmanial activity. This work consists in analyzing the structure of a validated target in *Leishmania*: the GDP-mannose pyrophosphorylase (GDP-MP), an enzyme involved in glycosylation and essential for amastigote survival. By comparing both human and *L. infantum* GDP-MP 3D homology models, we identified (i) a common motif of amino acids that binds to the mannose moiety of the substrate and, interestingly, (ii) a motif that is specific to the catalytic site of the parasite enzyme. This motif could then be used to design compounds that specifically inhibit the leishmanial GDP-MP, without any effect on the human homolog.

## Abstract (fr)

Les leishmanioses sont des parasitoses tropicales et subtropicales pour lesquelles les traitements classiques (*i.e.* antimoniés) sont toxiques et présentent des problèmes de résistance. Dans ce contexte, il est nécessaire de trouver de nouvelles séries chimiques ayant une activité antileishmanienne. Ce travail consiste à analyser la structure d’une cible validée chez *Leishmania* : la GDP-mannose pyrophosphorylase (GDP-MP), une enzyme impliquée dans la glycosylation et essentielle à la survie du stade amastigote. En comparant les modèles 3D par homologie des GDP-MP humaine et de *L. infantum*, nous avons identifié (i) un motif d’acides aminés commun qui interagit avec la partie mannose du substrat et (ii) un motif spécifique au site catalytique de l’enzyme du parasite. Ce motif pourrait être utilisé pour concevoir des composés qui inhibent spécifiquement la GDP-MP leishmanienne, tout en n’ayant aucun effet sur l’homologue humaine.

## Introduction

Leishmaniasis is caused by the protozoan parasite *Leishmania* spp. and is transmitted by the insect vector belonging to the Phlebotominae sub-family. This parasite grows within the sandfly as the motile promastigote form which is injected during blood meal in the host organism to transform into the intracellular amastigote form which divides within macrophages. Leishmaniases show various clinical manifestations: visceral, which is lethal in the absence of treatment, cutaneous and muco-cutaneous, for which there is no safe therapy currently. Several species of *Leishmania* are known to give rise to visceral (*L. donovani* or *L. infantum*), cutaneous (*L. major* or *L. mexicana*), or muco-cutaneous (*L. braziliensis*) leishmaniases.

In Europe, leishmaniasis is present all along the Mediterranean border. *Leishmania infantum* is the most common parasite responsible for human visceral leishmaniasis in the Mediterranean basin. The preferential hosts of this parasite are either immunocompetent children, human immunodeficiency virus (HIV) infected patients or dogs. These last years, a territorial expansion of leishmaniasis has been observed, possibly due to climate warming.

The first drugs that have been used for up to 60 years against leishmaniasis are antimonials. These molecules are highly toxic and increasingly ineffective due to the development of resistance. Moreover, oral miltefosine and injectable AmBisome^®^ (liposomal amphotericin B), which both produce side effects, now show some risks of drug resistance as well (Croft *et al.*, 2006). In this context, it becomes crucial to develop new treatments against leishmaniasis.

The present study relies on the structural analysis of a target involved in host-parasite interactions. These interactions are based on *Leishmania* glycoconjugates recognition by macrophages, allowing parasite internalization and intracellular development. Therefore, glycosylation pathway is a key-point in macrophage infection.

**Fig. 1.**
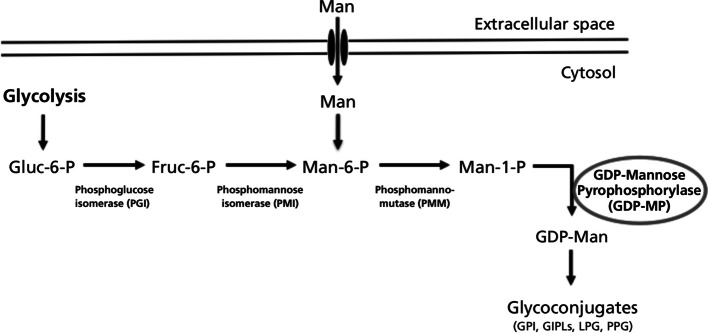
Mannose activation pathways and glycoconjugate biosynthesis in *Leishmania*. This pathway is used to synthesize various glycoconjugates: GPI (glycophosphatidylinositol) anchors, GIPLs (glycosylinositolphospholipids), LPG (lipophosphoglycans) or PPG (proteophosphoglycans). The GDP-MP enzyme is circled in grey.

Eukaryotic glycosylation involves many types of mannose- containing glycoconjugates, like N- or O-glycosylated proteins, glycolipids, or glycosylphosphatidylinositol (GPI) protein membrane anchors, which have important functions in a broad range of biological processes including intercellular adhesion or signaling (Varki, 2007). *Leishmania* synthesizes a range of mannose-rich glycoconjugates that are considered to be essential for parasite virulence (see for review Descoteaux & Turco, 1999). In particular, this protozoan parasite produces large amounts of unusual mannosylated cell-surface associated glycoconjugates (Fig. 1), such as lipophosphoglycans (LPG), proteophosphoglycans (PPG) or glycosylinositolphospholipids (GIPLs).

A prerequisite for biosynthesis of these glycoconjugates is the conversion of monosaccharides to the activated mannose, GDP-mannose. This nucleotide sugar is used as a mannose donor for all mannosylation reactions. In eukaryotic cells, mannose can either be internalized from the extracellular medium by means of a membrane transporter or originate from the action of the phosphomannose isomerase (PMI) on fructose-6-phosphate to produce mannose-6-P. This phosphorylated hexose is then converted in mannose-1-phosphate by the action of phosphomannomutase (PMM; Fig. 1). The production of GDP-mannose is then catalyzed by the GDP-mannose pyrophosphorylase (GDP-MP) according to the following reversible reaction:

Mannose-1-P + GTP ↔ GDP-mannose + PPi

Deletion of the gene encoding GDP-MP in *L. mexicana* is critical for amastigote survival *in vitro* and leads to a total loss of virulence *in vivo*, indicating that GDP-MP is an ideal drug target (Garami & Ilg, 2001; Stewart *et al.*, 2005). A high-throughput screening (HTS) assay, using a library of about 80,000 compounds, has recently been set up in order to select GDP-MP inhibitors (Lackovic *et al.*, 2010). From this study, some inhibitors exhibited an *in vitro* activity on *Leishmania* GDP-MP and on intracellular parasite proliferation confirming the relevance of this target for drug design.

These encouraging data prompted us to develop inhibitors specifically active on *L. infantum* GDP-MP from a rational approach relying on the comparative analysis of the leishmanial and human enzyme 3D structures. In this work, we will exclusively use the human isoform (β2) that shows the best homology to the leishmanial GDP-MP in order to develop the more specific possible compounds to the parasite enzyme. This study has been performed on the agent of human and canine leishmaniasis, *L. infantum*, which causes a growing health concern in many Mediterranean countries nowadays. Accordingly, this work should allow us to characterize compounds that could be active on both canine and human visceral leishmaniases.

## Materials and Methods

### Sequence alignments

The amino acid sequences of *L. infantum* (clone JPCM5 (MCAN/ES/98/LLM-877)), and human GDP-MPs were retrieved from the Genebank database (accession numbers: CAM68115.1 and NP_068806.1, respectively). Sequence from glucose- 1-phosphate thymidylyltransferase from *Sulfolobus tokodaii* was obtained from the Protein Data Bank (PDB). Sequence alignments were performed using the programme ClustalW2 (Larkin *et al.*, 2007). A slow pairwise alignment using BLOSUM matrix series (Henikoff & Henikoff, 1992) and a gap opening penalty of 15.0 were chosen for aligning the amino acid sequences. These alignments were further visualized using the programme Genedoc (version 2.7.000). The amino acids of the glucose-1-phosphate thymidylyltransferase (2ggo) active site were determined on the following website: http://www.ncbi.nlm.nih.gov/Structure/cdd/ cddsrv.cgi.

### Homology modelling

In a first step, we developed an automatic 3D model of *L. infantum* GDP-MP based on structural homologies with a template protein using the programme Modeller (version 9.9) on the ModWeb server (http:// modbase.compbio.ucsf.edu/ModWeb20-html/modweb. html), a server for protein structure modelling (Marti-Renom *et al.*, 2000). This 3D model was further used in the programme Sybyl (Tripos International, S.L., Missouri, USA) with the sequence alignment of both human and *L. infantum* GDP-MPs to manually build the human GDP-MP 3D model. Both leishmanial and human GDP-MP 3D models were then refined by classical techniques of molecular mechanics using AMBER (version 8.0; Case *et al.*, 2004). Each GDP-MP enzyme was minimized with AMBER 8.0 using the AMBER03 force field to relax the structure and to remove steric bumps. The minimizations were carried out by 2,000 steps of steepest descent followed by conjugate gradient minimization until the rms gradient of the potential energy was lower than 0.05 kcal.molÅ^−1^. A twin cut-off (10.0, 15.0 Å) was used to calculate non-bonded electrostatic interactions at every minimization step, and the non-bonded pair list was updated every 25 steps. A distance-dependent (e = 4r) dielectric function was used. The stereochemistry parameters of quality (Ramachandran plots) were also checked after minimization.

Molecular dynamics were used to explore the conformational spaces and search for the lowest energy conformations in our 3D models. The best model of both human and *L. infantum* GDP-MPs was then selected for structural analyses.

### Docking analysis

Docking analyses were performed without any constraints with 20 poses using the programme GOLD (version 5.0; Jones *et al.*, 1997). For each of the 20 independent genetic algorithm (GA) runs, a maximum number of 1,000 GA operations was performed on a single population of 50 individuals. Operator weights for crossover, mutation, and migration were set to 100, 100, and 0, respectively.

To allow poor nonbonded contacts at the start of each GA run, the maximum distance between hydrogen donors and fitting points was set to 5 Å, and nonbonded van der Waals energies were cut off at a value equal to kij (well depth of the van der Waals energy for the atom pair i,j).

## Results and Discussion

The GDP-MP enzyme has been proved to be essential for *Leishmania* amastigote survival and thus represents an ideal therapeutic target. If some GDP-MP inhibitors have already been characterized in *L. mexicana* (Lackovic *et al.*, 2010), their mode of inhibition and especially their specific action on the parasite’s enzyme remains unclear. In the present work, we report a structural comparison of both human and *L. infantum* GDP-MP 3D homology models which will be the rational basis to design compounds that specifically inhibit *L. infantum* GDP-MP and not the human homologous enzyme.

### 3D homology models of human and *L. infantum* GDP-MPs

Based on the *L. mexicana* GDP-MP structural homology with the uridyltransferase Glmu from *Streptococcus pneumoniae* and thymidylyltransferase from *Pseudomonas aeruginosa*, a 3D model of the GDP-MP from *L. mexicana* has been previously generated (Perugini *et al.*, 2005). However, no data was reported concerning the position of the catalytic site in this model.

Nowadays, GDP-MP crystal structures are only available in two thermophilic bacterial species: one in *Thermotoga maritima*, crystallized alone or with the substrates mannose-1-P, GTP or GDP-mannose (Protein Data bank (PDB) codes: 2x5s, 2x65, 2x60 and 2x5z; Pelissier *et al.*, 2010), and one in *Thermus thermophilus*, deposited in PDB in 2005 (PDB code: 2cu2). This last crystal was used as a template to generate a GDP-MP 3D model bound to its substrates in the pathogenic bacterium *Leptospira interrogans* (Asencion Diez *et al.*, 2010). These studies provide a detailed description of the enzyme active site along with conformational changes associated with ligand binding. Since no crystallographic structure is currently available for human and *L. infantum* GDP-MPs, we have generated homology models of these enzymes to develop pharmacomodulated compounds based on a rational analysis of GDP-MP catalytic sites.

**Fig. 2.**
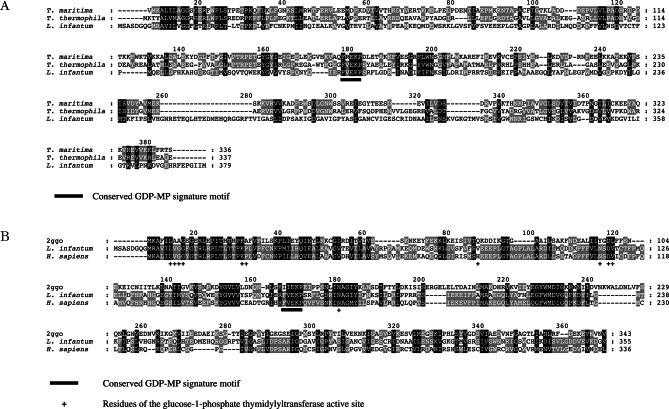
Sequence alignment of GDP-MPs. A. Multiple sequence alignment of *L. infantum*, *T. thermophilus*, and *T. maritima* GDP-MPs. The *L. infantum* GDP-MP presents 16.1% and 15.4% of identity with the *T. thermophilus* and *T. maritima*, respectively. The signature motif F(V)EKP, essential for the activity of GDP-MPs, is underlined. B. Multiple sequence alignment of both human and *L. infantum* GDP-MPs with the protein used as a template for 3D modelling (glucose-1-phosphate thymidylyltransferase from *Sulfolobus tokodaii*: 2ggo). This alignment shows that *L. infantum* and human GDP-MPs present 29% and 25% of identity with the template protein, respectively. Both human and leishmanial enzymes share 49% of identity. The amino acids of the template protein active site were identified as described in Materials & Methods. The GDP-MP signature motif F(V)EKP (underlined) is present in both human and leishmanial enzymes, but not in the template protein.

The analysis of multiple sequence alignments showed us that the *L. infantum* GDP-MP presents a higher identity score with the homologous enzyme in *T. thermophilus* (16.1%) than in *T. maritima* (15.4%; Fig. 2A). Taking into account that a sequence identity of at least 20-25% is required to build a reliable homology model, this sequence alignment seems to present too much variations to build a reasonable 3D model of *L. infantum* GDP-MP.

**Fig. 3.**
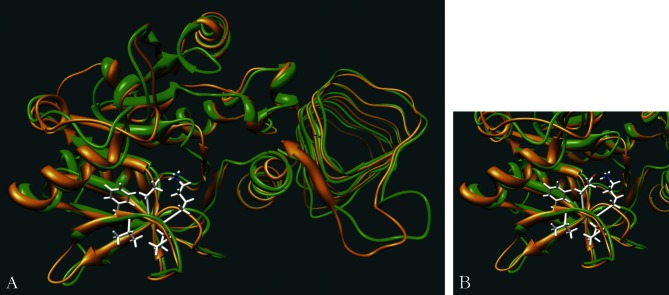
3D model of *L. infantum* GDP-MP. A. Superposition of the crystallized *Sulfolobus tokodaii* glucose-1-phosphate thymidylyltransferase (ribbon-tube in orange) and the 3D model of the *L. infantum* GDP-MP (ribbon-tube in green). B. Detailed position of the conserved motif F(V)EKP (in sticks) in *L. infantum* GDP-MP.

Two complementary approaches can be used to build a 3D homology model: a/ using the programme Modeller, b/using the Biopolymer module of the Sybyl suite software. Using the programme Modeller with the *L. infantum* GDP-MP sequence as an input, we obtained a 3D model based on structural homology with the following template protein: the glucose- 1-phosphate thymidylyltransferase from *Sulfolobus tokodaii* (PDB code : 2ggo; Fig. 3A). Although this protein belongs to the same enzyme family as GDP-MPs, the NTP transferases, the amino acids constituting its active site are different from GDP-MPs since it does not include notably the signature motif F(V)EKP which is essential for GDP-MP activity (Fig. 2B). However, this motif is present in a region that is structurally conserved in the template protein (Fig. 3B). According to the programme Modeller, the best selected 3D homology model was generated between amino acids 9 and 355 of the *L. infantum* GDP-MP (379 amino acids in total). In this interval, the *L. infantum* GDP-MP and the template protein from *S. tokodaii* present 29% of identity and 47% of similarity (Fig. 2B), giving a good credit to the selected homology 3D model.

**Fig. 4.**
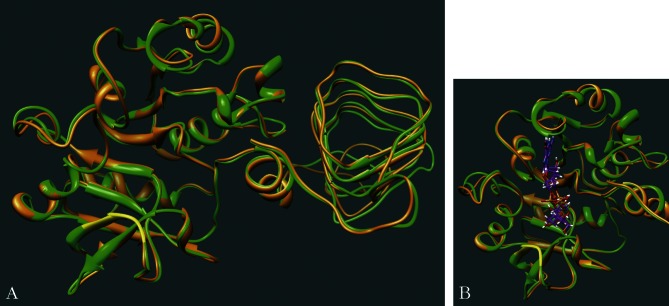
Comparison of human and *L. infantum* GDP-MP models. A. Superposition of human and *L. infantum* GDP-MP 3D models. Ribbon-tubes in green and orange represent the *L. infantum* and the human GDP-MPs, respectively. The F(V)EKP conserved motif is colored in yellow. B. Position of the GDP-mannose in both human and *L. infantum* GDP-MPs. This figure represents the superposition of both human and *L. infantum* GDP-MPs including the substrate GDP-mannose (purple). The position of the GDP-mannose was determined by docking. Ribbon-tubes in green and orange represent the *L. infantum* and the human GDP-MPs, respectively. The F(V)EKP conserved motif is colored in yellow.

Using the alignment of both human and leishmanial GDP-MPs (49% identity; Fig. 2B) and the *L. infantum* enzyme 3D model in the Sybyl programme, we built a 3D model of the human GDP-MP between amino acids 1 and 336 (360 amino acids in total). This model, as well as the 3D model of the *L. infantum* GDP-MP automatically generated by the programme Modeller, were further refined using the programme AMBER in order to minimize the conformational energies of the models and therefore stabilize the protein structures. Fig. 4A shows that both *L. infantum* and human GDP-MP 3D models are structurally very similar.

### Docking analyses of the GDP-mannose on human and *L. infantum* GDP-MPs

In order to determine the position of the substrate GDP-mannose (the largest GDP-MP substrate) in the GDP-MP catalytic site, we performed docking analyses without any constraints on both human and *L. infantum* enzyme 3D models. These analyses revealed that the position of the substrate GDP-mannose is similar in both GDP-MP models (Fig. 4B), confirming the reliability of these GDP-MP 3D models.

**Fig. 5.**
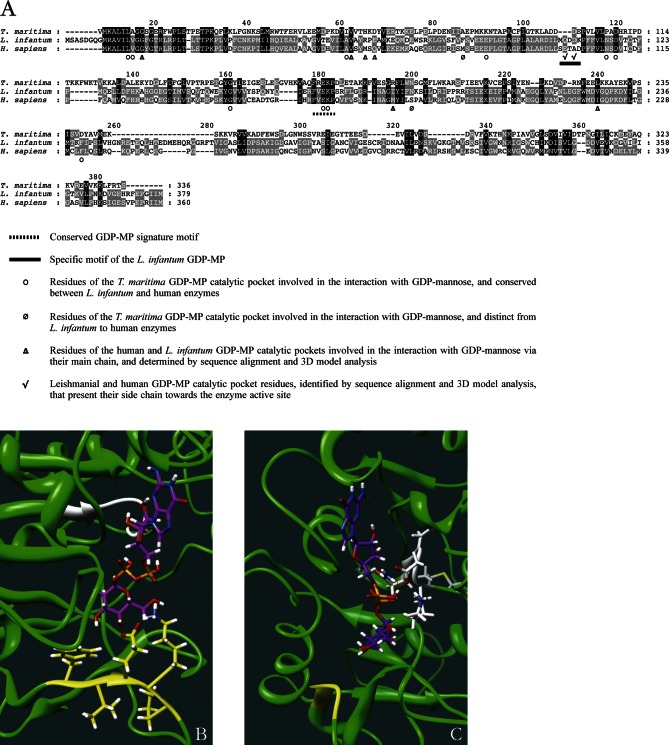
Identification of a specific motif in the GDP-MP of *L. infantum*. A. Multiple sequence alignment of *T. maritima*, *L. infantum* and human GDP-MPs. The specific motif of the *L. infantum* GDP-MP catalytic pocket is underlined with a full line. Note the presence of the signature motif F(V)EKP (underlined with a dashed line) in these three enzymes. B. Interactions mediated between GDP-mannose (purple) and the F(V)EKP conserved motif of the *L. infantum* GDP-MP (green). The amino acids of the conserved motif (yellow) are represented in sitck. The red lines represent H-bonds. The specific motif is colored in white. C. Interactions mediated between GDP-mannose (purple) and the specific motif of the *L. infantum* GDP-MP (green). The amino acids of the specific motif (white) are represented in stick. The red lines represent H-bonds. The F(V)EKP conserved motif is colored in yellow.

The study of these docking data together with multiple sequence alignments allowed us to delineate both human and leishmanial enzyme catalytic sites. The alignment of the GDP-MPs from human, *L. infantum* and *T. maritima* showed that most of the residues of the bacterial enzyme active site are conserved between *L. infantum* and human, especially the E and K residues of the F(V)EKP motif which bind to the mannose and phosphate moieties of the substrate, respectively (Pelissier *et al.*, 2010; Fig. 5A, B). Only few residues of the bacterial GDP-MP catalytic pocket are distinct from *L. infantum* to human (Fig. 5A). However, the docking analysis revealed that these specific residues are not conserved in the active site of both human and leishmanial enzymes.

Futhermore, we used the alignment of both human and *L. infantum* GDP-MP sequences and our docking data to identify new specific residues in the catalytic pockets of both human and leishmanial enzymes (Fig. 5A). Among these residues, we selected those which have their side chain directed towards the catalytic pocket of the enzyme. In this way, we identified a motif of amino acids that is specific to *L. infantum* (M106, Q107, D108, D109 and K110) compared to human (S98, E99, T100, A101 and D102). This motif is not found in the catalytic pocket of the *T. maritima* GDP-MP crystal (Pelissier *et al.*, 2010). In the *L. infantum* GDP-MP specific motif, both Q107 and D109 mediate H-bonds with the GDP moiety of the GDP-mannose (Fig. 5C). Taking into account that inhibitor interactions should be specific to the leishmanial enzyme, drug design will further focus on the selective Q107 and D109 amino acids of the *L. infantum* GDP-MP in order to specifically inhibit this enzyme, and not the human homolog.

## Conclusion

In conclusion, this study allowed us to define in the *L. infantum* GDP-MP sequence a common region that binds to the mannose moiety of the substrate, and a selective region, compared to the human homolog, that seems to be promising for specific drug design. This comparative *in silico* analysis of both *L. infantum* and human GDP-MPs will be followed by the design of inhibitors and further experimental validations using enzyme assays. The specificity of these inhibitors will be further validated both *in vitro* and *in vivo* on *L. infantum* infected macrophages and mice, respectively. We expect from this study to develop new compounds active against drug-resistant parasites.
